# Diagnosis and management of type 2 diabetes mellitus in patients with ischaemic heart disease and acute coronary syndromes - a review of evidence and recommendations

**DOI:** 10.3389/fendo.2024.1499681

**Published:** 2025-01-22

**Authors:** Muhammad Usman Shah, Alun Roebuck, Bala Srinivasan, Joanna Kate Ward, Paul Edward Squires, Claire Elizabeth Hills, Kelvin Lee

**Affiliations:** ^1^ Cardiorenal Group, Diabetes, Metabolism, & Inflammation, Joseph Bank Laboratories, University of Lincoln, Lincoln, United Kingdom; ^2^ Lincoln Heart Centre, United Lincolnshire Hospitals, Lincoln, United Kingdom; ^3^ Department of Diabetes and Endocrinology, United Lincolnshire Hospitals, Lincoln, United Kingdom

**Keywords:** type 2 diabetes mellitus, ischaemic heart disease (IHD), acute coronary syndrome, management optimisation, diagnosis

## Abstract

Type 2 diabetes mellitus (T2DM) represents a major healthcare condition of the 21st century. It is characterised by persistently elevated blood glucose occurring as a result of peripheral insulin resistance and reduced insulin production which may lead to multiple long-term health conditions such as retinopathy, neuropathy, and nephropathy. The estimated number of individuals suffering from diabetes mellitus (DM) is expected to rise to 591 million by the year 2035 with 4.4 million in the United Kingdom (UK) alone, 90% of which is attributed to T2DM. Moreover, a significant proportion of individuals may have undetected diabetes mellitus, especially among those presenting with symptoms of ischaemic heart disease (IHD). This is particularly important in those individuals presenting with acute coronary syndromes (ACS) who are at the highest risk of complications and sudden cardiac death. Identifying abnormal levels of common biochemical markers of diabetes, such as capillary blood glucose or glycated haemoglobin (HbA1c) in these patients is important for early diagnosis, which will then allow for timely intervention to improve outcomes. However, a significant proportion of individuals who meet the criteria for the diagnosis of diabetes remain undiagnosed, representing missed opportunities for early intervention. This may result in a prolonged period of untreated hyperglycaemia, which can result resulting in significant further microvascular and macrovascular complications. There is an increased risk of IHD, heart failure, cerebrovascular accidents (CVA), and peripheral artery disease (PVD). These account accounting for 50% of deaths in patients with T2DM. Cardiovascular diseases in the context of diabetes particular represent a significant cause of morbidity and mortality with a two to three times higher risk of cardiovascular disease in individuals with T2DM than in those without the condition normo-glycaemia. In the United Kingdom UK alone, around 120 amputations, 770 CVA, 590 heart attacks, and more than 2300 presentations with heart failure per week are attributed to diabetes DM. with One 1 in six 6 hospital beds and around 10% of the healthcare budget may be being spent on managing diabetes DM or its complications. Therefore, it represents a significant burden on our healthcare system.

## 1 Introduction

Type 2 diabetes mellitus (T2DM) is characterised by persistently elevated blood glucose occurring as a result of peripheral insulin resistance and reduced insulin production (1) which may lead to multiple long-term health conditions such as retinopathy, neuropathy, and nephropathy (2). A global healthcare concern, the estimated number of individuals suffering from diabetes mellitus (DM) is expected to rise to 591 million by the year 2035 (3) with 4.4 million in the United Kingdom (UK) alone (4), 90% of which is attributed to T2DM (3, 4). Moreover, a significant proportion of individuals may have undetected diabetes mellitus (3–5), especially among those presenting with symptoms of ischaemic heart disease (IHD) (6–8). This is particularly important in those individuals presenting with acute coronary syndromes (ACS) who are at the highest risk of complications and sudden cardiac death (9). A significant proportion of individuals remain undiagnosed, representing missed opportunities for early intervention (6). This may result in a prolonged period of untreated hyperglycaemia, resulting in significant microvascular and macrovascular complications (3) accounting for 50% of deaths in patients with T2DM (1). Cardiovascular diseases in particular represent a significant cause of morbidity and mortality with a two to three times higher risk of cardiovascular disease in individuals with T2DM than in those without the condition (1). In the UK alone, around 120 amputations, 770 CVA, 590 heart attacks, and more than 2300 presentations with heart failure per week are attributed to DM (4) with 1 in 6 hospital beds and 10% of the healthcare budget being spent on managing DM or its complications (4). Therefore, it represents a significant burden on our healthcare system (6).

The management of T2DM with cardiovascular and renal disease has changed significantly. Over the last 10 years, landmark cardiovascular trials have demonstrated significant benefits with certain groups of glucose-lowering medications, including sodium-glucose co-transport-2 (SGLT2) inhibitors and glucagon-like peptide-1 receptor agonists (GLP-1RA), transforming recommendations and treatment options for those with DM (10, 11). Individuals with new or established IHD, especially those with acute coronary syndromes, represent the group with the highest risk of further cardiovascular events (10). The above-mentioned therapies not only reduce the risk of further cardiovascular events (12–16) but also reduce renal complications (13, 17–20) and therefore, would be most useful in patients with T2DM and underlying cardio-renal syndrome. However, the trials above were performed among participants with established stable IHD and not in ACS. Dedicated randomised controlled trials in patients with DM to assess efficacy and safety with regards to cardiovascular outcomes and mortality in ACS have not yet been performed, although registry data suggests a significant reduction in hospitalisation for heart failure and death (21). Whilst the safety profile of these medicines is well established in patients with stable disease, concerns may arise when used in ACS. These pertain to not only the development of diabetic complications such as diabetic ketoacidosis (DKA) and hypoglycaemia, but also of side effects arising from concomitant cardiovascular medicine use such as angiotensin-converting enzyme inhibitors (ACEi) and beta-blockers that may result in symptomatic hypovolemia, which may be detrimental in older adult individuals (22). Previous studies have shown very strict glycaemic control to result in increased mortality in those with advanced T2DM and at high risk of cardiovascular disease (23), whilst others have shown no net benefit of cardiovascular medicines such as angiotensin receptor–neprilysin inhibitor (ARNI) in an ACS setting (24). Therefore, it is important for all clinicians, especially cardiologists and diabetologists, to be familiar with these developments and ensure patients are appropriately prescribed medications with proven cardiovascular benefits. Moreover, it is also important to ensure that patients are assessed and screened adequately to diagnose the condition early. This combination of early screening, correct diagnosis, and optimal medical therapy would help further reduce the likelihood and incidence of cardiovascular complications, morbidity, and mortality (10). In this review, we will be examining topical evidence in the diagnosis and management of T2DM in the setting of IHD, particularly ACS, assessing key recommendations from the latest clinical trials as well as covering challenges that clinicians may face, to help develop local protocols for optimal care of these high-risk individuals.

## 2 Diagnosis and monitoring

**Figure 1:**
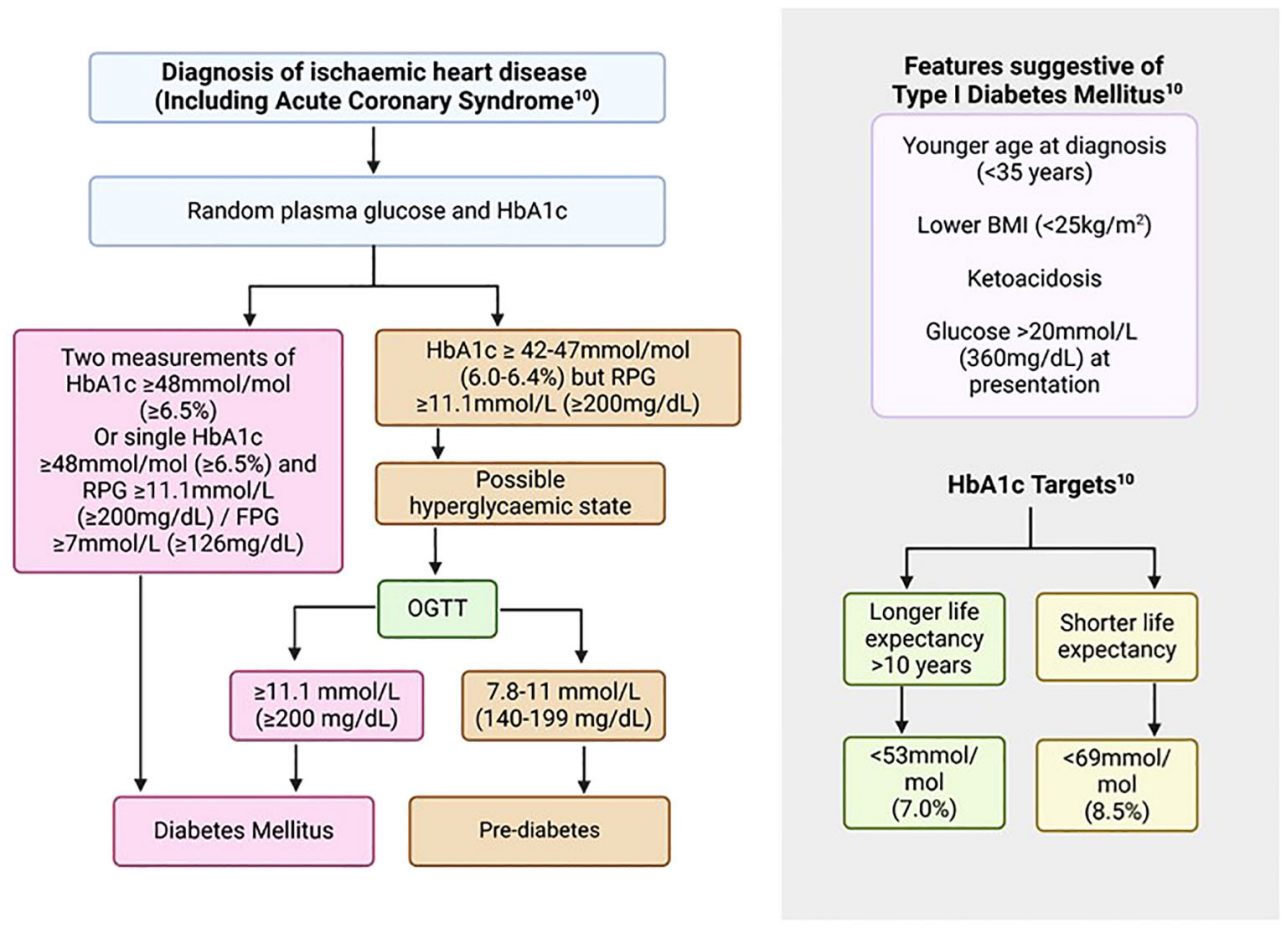
Screening and diagnosis of diabetes mellitus in patients with ASCVD (10). ASCVD, Atherosclerotic cardiovascular disease; BMI, Body mass index; FPG, Fasting plasma glucose; HbA1c, Glycated haemoglobin; OGTT, Oral glucose tolerance test; RPG, Random plasma glucose.

Diabetic range readings for biochemical markers, including fasting plasma glucose (FPG) and glycated haemoglobin (HbA1c), were traditionally based on the degree of hyperglycaemia that would result in the first development of non-proliferative retinopathy (25). In the presence of typical features of T2DM such as polyuria and gradual weight loss, or development of cardiovascular complications such as ACS, one measurement of a biochemical marker in the diabetic range would be required to reach the diagnosis. In the absence of typical features, at least two measurements in the diabetic range would be needed for this purpose (10). Traditionally, FPG and oral glucose tolerant test (OGTT) were used for screening and diagnostic purposes. However, other biomarkers have been developed which aim to address some of the limitations of these measures, the most commonly adopted one being HbA1c (26), summarised in **Table 1** and **Figure 1**. Following confirmation of diagnosis, reduction in HbA1c levels to less than 53 mmol/mol (7%) decreases microvascular complications. However, the effects on macrovascular conditions are more varied with effects becoming apparent between 6.5 to 10 years (10). Very low glucose levels may be associated with worse outcomes. Therefore, balance is required and targets should be personalised for individuals based on their life expectancy and underlying conditions with a more strict target (<53 mmol/mol, 7%) for younger individuals with a life expectancy of at least 6-10 years and a more relaxed target (<69mmol/mol, 8.5%) for those with shorter life expectancy (10).

**Table 1:** Comparison of hyperglycaemic markers (10, 26).

Marker	Time assessed	Normal range	Diabetic range	Diagnostic utility	Monitoring Utility	Target level for optimal diabetes control	Advantages	Limitations
FPG	Real-time	ADA:<5.6 mmol/L (100 mg/dl) WHO:<6.0 mmol/L (110 mg/dl)	≥7.0 mmol/L (126 mg/dl)	+	+	–	-Instantaneous assessment -Easy to perform	-Affected by illness and stress -Fasting state required
OGTT/RPG	Real-time	≤7.7 mmol/L (139 mg/dl)	≥11.1 mmol/L (200 mg/dl)	+	–	6-10 mmol/L (108-180 mg/dl)	-Instantaneous assessment -Helpful in situations of diagnostic uncertainty	-Cumbersome -Significant variations dependent on stress and illnesses
HbA1c	2-3 months	ADA: <39 mmol/mol (5.7%) WHO: <42 mmol/mol (6.0%)	≥48 mmol/mol (6.5%)	+	+	Longer life expectancy <53 mmol/mol (7.0%) Shorter life expectancy <69 mmol/mol (8.5%)	-Standardised -Low variability -No fasting state requirements	Affected by underlying blood disorders and anaemia
Fructosamine	2-3 weeks	194.8-258.0 umol/L	>287 umol/L	–	+	Longer life expectancy <317 umol/L Shorter life expectancy <405 umol/L	-No fasting state requirement -Not affected by haemoglobinopathies or anaemia -Strong correlation with HbA1c -Marker of choice in advanced CKD	-Lack of standardisation with higher variability -Affected by conditions resulting in low proteins and thyroid dysfunction
Glycated albumin	2-3 weeks	10.7%-15.1%	≥ 15.5%	–	+	Longer life expectancy < 18.1% Shorter life expectancy <25.2%	-No fasting state requirement -Not affected by haemoglobinopathies or anaemia	-Lack of standardisation with higher variability -Affected by conditions resulting in low proteins and thyroid dysfunction
1,5-AG(120)	1-2 weeks	14.4-30.2 µg/ml	–	–	+	≥ 14.0 µg/ml	-No fasting state requirement -Useful in the detection of glycaemic excursions	-Unreliable in conditions including CKD, pregnancy, and renal replacement therapy -Unreliable when taking SGLT2is

Summary of glycaemic markers for the diagnosis and monitoring of diabetes mellitus. ADA, American Diabetes Association; AG, Anhydroglucitol; FPG, Fasting Plasma Glucose; HbA1c, Glycated Haemoglobin; OGTT, Oral Glucose Tolerance Test; RPG, Random Plasma Glucose; WHO, World Health Organisation.

The Myocardial Infarction National Audit Project (MINAP), a registry of all ACS events in the United Kingdom (UK), reports 21% of patients to have had pre-existing diabetes (27) while others report a higher proportion of 25-30% (28). However, it is estimated that between 2-4% of individuals may have undiagnosed diabetes mellitus (29). The UK National Institute for Health and Care Excellence (NICE) recommends baseline glucose levels in all individuals presenting with ACS and follow-on FBG or HbA1c only if hyperglycaemia (glucose >11.0 mmol/litre) is detected in those with no previous history of diabetes mellitus (DM) (30). However, not all patients with diabetes mellitus will develop hyperglycaemia during the admission and this approach will miss those patients who have undiagnosed DM. Moreover, an HbA1c measurement is critical in the assessment for diabetes control in those with previously diagnosed DM when admitted with an ACS. Therefore, the European Society of Cardiology (ESC) guidelines recommend HbA1c or FBG for screening and HbA1c for further monitoring of DM (10). These tests have advantages and limitations which have to be taken into account when managing individuals with DM and ACS and are discussed below.

### 2.1 Fasting plasma glucose

Fasting plasma glucose (FPG) is a direct measure of hyperglycaemia. This represents a convenient and quick method for the diagnosis and monitoring of diabetes. Blood glucose levels are measured following an 8-hour fast of no caloric intake (10, 25, 26). Benefits include assessment of the real-time glycaemic status, as well as convenience and ready availability. However, it is limited by variations during acute illnesses and stress, including ACS and myocardial infarction, which may affect the accuracy of this test (26).

### 2.2 Two-hour oral glucose test tolerance

Seventy-five grams of glucose is administered orally in a resting state followed by an assessment of plasma glucose, two hours after ingestion (26). This can be particularly helpful in unclear situations where other markers of diabetes may be affected due to underlying comorbid conditions (10). However, it is cumbersome, time-consuming, and may be affected by acute illnesses and stress, resulting in significant variation (10, 25, 26). A modified one-hour version of the OGTT is available however, further validation is required before this can be adopted in routine practice (10).

### 2.3 Glycated haemoglobin

Glycated haemoglobin (HbA1c) is produced in response to a post-translational modification and binding of haemoglobin with glucose. It measures the average hyperglycaemia over a two-to-three-month period and therefore, represents a more representative marker for diagnostic and monitoring purposes (26). Initially, clinical accuracy was limited by variation in the method of analysis, however, a subsequent standardisation programme by the International Federation of Clinical Chemistry (IFCC) led to the development of a comprehensive reference system based on various methods with excellent between-method correlation and reduction in variability between results (26). This was subsequently adopted by the Global Consensus with values reported in IFCC-related (mmol/mol) and the Diabetes Control and Complications Trial (DCCT, %) units (26). The main benefits of using HbA1c for diagnosis and monitoring of T2DM include ease of measurement, no fasting requirement, and standardisation with a strong correlation with complications of diabetes (10, 26). However, care must be taken in certain conditions which affect haemoglobin levels, resulting in false measurements. These include anaemia, haemoglobinopathies, pregnancy, and chronic renal failure (10, 26). Similarly, care must be taken in situations of stress hyperglycaemia with elevated glucose levels and normal HbA1c as this may be the first feature of recent-onset diabetes. In these situations, an OGTT is recommended to further assess glycaemic status (**Figure 1**) (26). Individuals with prediabetic ranges of HbA1c (42-47 mmol/mol, World Health Organisation criteria), who represent a significant proportion of patients presenting with ACS, are at higher risk of development of DM and should be advised on health behavioural changes including a balanced diet and exercise with annual screening for the condition (10, 30). Despite these caveats, the overall ease of performance, along with reproducibility and strong correlations with diabetic complications make it a good screening, diagnostic, and monitoring tool for the management of diabetes.

### 2.4 Fructosamine and glycated albumin

These represent glycated plasma proteins as a result of non-enzymatic glycation with circulating glucose molecules. In contrast to HbA1c which is a measure of the glycaemic control over a two-to-three month period, fructosamine and glycated albumin (GA) have a much shorter life span and therefore represent the glycaemic status over a 2-3 week period (26). With recent advancements and standardisations, these represent additional monitoring tools, especially in individuals with haemoglobinopathies, anaemia, and chronic kidney disease (CKD), including those on renal replacement therapy, where HbA1c may be inaccurate. In addition, it can provide a more timely assessment of diabetes control following any changes in management strategies. However, care has to be taken in those with underlying conditions that may affect protein and albumin turnover such as nephrotic syndrome, hypothyroidism, and chronic liver disease, where the values may be inaccurate (26).

### 2.5 1,5-Anhydroglucitol

This is a monosaccharide similar in structure to glucose. It is acquired mainly through the ingestion of food, with only a minimal amount produced by the body. It is excreted into urine where a majority is subsequently reabsorbed by sodium-glucose co-transporters. Reabsorption is competitively inhibited by glucose therefore, in states of hyperglycaemia with glucosuria, 1,5-AG is rapidly excreted in the urine, resulting in a reduction of levels in the body (26). It may take several weeks for the concentration to normalise. In individuals with labile glucose levels but otherwise normal HbA1c, 1,5-AG may be helpful to better characterise diabetes control (26). Similarly, this would also allow earlier assessment of response to specific pharmacological therapies. A fasting state is not required and is strongly correlated with retinopathy and CKD. Due to renal excretion, accuracy may be affected by CKD, renal replacement therapy, and the use of sodium-glucose co-transporter 2 (SGLT2) inhibitor therapy (26).

### 2.6 Recommendations

In individuals with new or established stable IHD or with an ACS, a HbA1c should be performed to screen for DM in those without a previous history of the condition. Individuals with known DM should have a HbA1c measurement for an up-to-date assessment of their DM control particularly in context of their IHD or ACS. In the context of an ACS, a single HbA1c and FBG/RBG value in diabetic range would fulfil the criteria for the diagnosis of DM. Clinical assessment, co-morbid conditions and family history would help with further differentiation into T1DM and T2DM. Those with features suggestive of T1DM such shorter duration of symptoms or younger age should be referred to the appropriate diabetic services for further anti-body testing (**Figure 1**). Individuals with T2DM are likely to have a more gradual onset of symptoms of DM, if any, and are likely to be older with a strong family history of the condition, and represent the vast majority of IHD patients with diabetes.

Achieving near normoglycaemia in individuals with DM leads to a reduction in the micro and macrovascular complications of the condition as shown in landmark trials such as the DCCT (Diabetes Control and Complications Trial) and the UKPDS (United Kingdom Prospective Diabetes Study) and their follow on meta-analyses (10). In contrast, observational data suggests increased mortality with very strict glycaemic control, highlighting the importance of avoiding hypoglycaemia (10, 31). Therefore, a pragmatic and individualized approach is required for each patient. Glycated haemoglobin (HbA1c) should be used for monitoring of diabetes control, aiming for <53 mmol/mol in younger individuals with a longer life expectancy whilst aiming for <69 mmol/mol in those with a life expectancy of less than 10 years (10). Care has to be taken in the presence of blood disorders such as anaemia and haemoglobinopathies which can affect HbA1c levels. In these circumstances, whilst limited by the lack of direct evidence assessing target values for diabetes control in those with IHD, nonetheless, we recommend the use of HbA1c equivalent diabetic ranges of FBG/RBG or OGTT for diagnostic (10) and age and prognosis dependent HbA1c target equivalent levels of Fructosamine (32) for monitoring purposes (**Table 1**).

In the setting of ACS, individuals may experience stress-related hyperglycaemia and, therefore, RBG/FBG and HbA1c should both be performed. Diabetes mellitus should be diagnosed if the HbA1c is in the diabetic range, however, if it is in the non-diabetic range with elevated RBG/FBG, then an OGTT should be performed in a staged manner to further assess for latent DM (**Figure 1**). Limitations to the use of HbA1c apply as above and where the results may be inaccurate due to underlying conditions, then FBG should be utilised as well.

## 3 Management

Managing T2DM in individuals with established or new IHD can be complex. It not only involves addressing uncontrolled hyperglycaemia to target levels where long-term complications may be reduced but also comprises early initiation of medications which are now proven to have cardiovascular benefits, irrespective of HbA1c levels (10). The latter includes classes of drugs such as SGLT2 inhibitors and GLP-1 receptor agonists (GLP1RAs) (**Table 2**) (10). In addition to therapeutic strategies, lifestyle modification and medication compliance are important factors that need to be addressed regularly (10). This represents a dynamic approach to tackling both conditions, avoiding management inertia, to achieve optimal results in the long term.

**Table 2:** Summary of major outcome trials.

Trial	Year	Therapeutic agent	Inclusion criteria	Number of participants	Primary outcomes	Results	HHF benefits	CVA Benefits	Renal benefits	Comparison of side effects between IMP and placebo
SGLT2is										
EMPA-REG OUTCOME Zinman et.al.	2015	Empagliflozin	T2DM and high risk of ASCVD	7020	Composite of death from cardiovascular causes, nonfatal myocardial infarction, or nonfatal stroke	Significant reduction in primary outcome (0.86, 0.74 to 0.99; p= 0.04)	Yes			More frequent genital infection (6.4% vs 1.8%) with Empagliflozin
CANVAS Neal et.al.	2017	Canagliflozin	T2DM and high risk of ASCVD	10142	Composite of death from cardiovascular causes, nonfatal myocardial infarction, or nonfatal stroke	Significant reduction in primary outcomes (hazard ratio, 0.86; 95% confidence interval, 0.75 to 0.97; p= 0.02)			Yes	Increased risk of amputations with Canagliflozin 6.3 vs. 3.4 (placebo) participants per 1000 patient-years
DECLARE–TIMI 58 Wiviott et.al.	2019	Dapagliflozin	T2DM and high risk of or established ASCVD	17160	Composite of death from cardiovascular causes, nonfatal myocardial infarction, or nonfatal stroke	Non-significant reduction in the primary outcome (hazard ratio, 0.93; 95% CI, 0.84 to 1.03; P=0.17)	Yes		Yes	Dapagliflozin associated with DKA (0.3% vs. 0.1% placebo, P=0.02) and genital infections (0.9% vs. 0.1% placebo, P<0.001)
CREDENCE Perkovic et.al.	2019	Canagliflozin	T2DM and Albuminuric CKD	4401	Composite of end-stage renal disease, doubling of serum creatinine, or death from renal or cardiovascular causes	Significant reduction in primary outcome hazard ratio, 0.70; 95% confidence interval, 0.59 to 0.82; P = 0.00001	Yes		Yes	No significant differences
DAPA-HF McMurray et.al.	2019	Dapagliflozin	Symptomatic heart failure with EF less than or equal to 40%	4744	Composite of cardiovascular death, HHF, or urgent visits for heart failure	Significant reduction in primary outcomes (hazard ratio, 0.74; 95% CI, 0.65 to 0.85; P<0.001)	Yes			No significant differences
EMPEROR-Reduced Packer et.al.	2020	Empagliflozin	Symptomatic heart failure with EF less than or equal to 40%	3730	Composite of cardiovascular death or HHF	Significant reduction in primary outcome (hazard ratio 0.75; 95% confidence interval, 0.65 to 0.86; P<0.001)	Yes		Yes	More frequent genital infection with Empagliflozin
SCORED Bhatt et.al.	2020	Sotagliflozin	T2DM and CKD (eGFR 25-60 ml/min/1.73m^2^	10584	Composite of CV death and HHF (Including urgent visits)	Significant reduction in the primary outcome (hazard ratio, 0.74; 95% confidence interval, 0.63 to 0.88; P<0.001)	Yes			Sotagliflozin associated with GI Symptoms Genital infections Volume depletion DKA
DAPA-CKD Heerspink et.al.	2020	Dapagliflozin	Albuminuric CKD	4304	Composite of sustained 50% decline in GFR, ESRD, or death from renal or cardiovascular causes	Significant reduction in primary outcome (hazard ratio, 0.61; 95% confidence interval, 0.51 to 0.72)			Yes	No significant differences
VERTIS CV Cannon et.al.	2020	Ertugliflozin	T2DM and ASCVD	8246	Composite of death from cardiovascular causes, nonfatal myocardial infarction, or nonfatal stroke	No significant reduction in the primary outcome (hazard ratio, 0.97; 95.6% confidence interval, 0.85 to 1.11)				Ertugliflozin associated with amputations (2.1% vs 1.6% placebo) and DKA (0.4% vs 0.1% placebo)
SOLOIST-WHF Bhatt et.al.	2020	Sotagliflozin	T2DM with recent HHF event	1222	Composite of cardiovascular death, HHF, or urgent visits for heart failure	Reduction in the primary outcome (hazard ratio, 0.67; 95% confidence interval, 0.52 to 0.85; P<0.001)	Yes			Diarrhoea (6.1% vs 3.4% placebo) and hypoglycaemia (1.5% vs 0.3% placebo) were more frequent in patients with Sotagliflozin
EMPEROR-Preserved Anker et.al.	2021	Empagliflozin	Symptomatic heart failure with EF>40%	5988	Composite of cardiovascular death or HHF	Significant reduction in primary outcome with Empagliflozin (hazard ratio, 0.79; 95% confidence interval, 0.69 to 0.90; P<0.001)	Yes			Uncomplicated Urinary tract and genital infections and hypovolemia more common in the Empagliflozin group
DELIVER Solomon et.al.	2022	Dapagliflozin	Symptomatic heart failure with EF>40%	6263	Composite of cardiovascular death, HHF, or urgent visits for heart failure	Significant reduction in the primary outcome (hazard ratio, 0.82; 95% confidence interval [CI], 0.73 to 0.92; P<0.001)	Yes			No significant differences
EMPULSE Voors et.al.	2022	Empagliflozin	Acute heart failure irrespective of EF	530	hierarchical composite of death from any cause, HHF events and time to first HHF, or a 5 point or greater difference in change from baseline in the KCCQ Total Symptom Score at 90 days, as assessed using a win ratio	Stratified win ratio, 1.36; 95% confidence interval, 1.09–1.68; P = 0.0054 for Empagliflozin	Yes			No significant differences
EMPA-KIDNEY Herrington et.al.	2022	Empagliflozin	Albuminuric CKD (Patients with GFR 20-45 ml/min/1.73m^2^ did not require the presence of albuminuria)	6609	Composite of sustained at least 40% decline in GFR, ESRD, or death from renal or cardiovascular causes	Significant reduction in the primary outcome (hazard ratio, 0.72; 95% confidence interval CI, 0.64 to 0.82; P<0.001)	Yes		Yes	DKA and lower limb amputations more common with Empagliflozin
DAPA-MI James et.al.	2023	Dapagliflozin	MI (Excluding patients with diabetes)	4017	Composite of cardiovascular death or HHF	No significant reduction in key cardiovascular outcomes (hazard ratio, 0.95; 95% CI, 0.64 to 1.40)				No significant differences
EMPACT-MI Butler et.al.	2024	Empagliflozin	MI (with or without T2DM)	3260	Composite of all-cause death or HHF	No significant reduction in the primary outcome (hazard ratio, 0.90; 95% confidence interval, 0.76 to 1.06; P=0.21)				No significant differences
GLP1RAs										
ELIXA Pfeffer et.al.	2015	Lixisenatide	T2DM and recent ACS	6068	Composite of cardiovascular death, MI, stroke or HUA	No significant reduction in the primary outcome (Hazard ratio, 1.02; 95% confidence interval [CI], 0.89 to 1.17)				No significant differences
SUSTAIN-6 Marso et.al.	2016	Semaglutide	T2DM	3297	Composite of death from cardiovascular causes, nonfatal myocardial infarction, or nonfatal stroke	Significant reduction in the primary outcome (hazard ratio, 0.74; 95% confidence interval [CI], 0.58 to 0.95)		Yes	Yes	Semaglutide was associated with a significantly higher incidence of Vitreous haemorrhage, blindness or conditions requiring further intervention
LEADER Marso et.al.	2016	Liraglutide	T2DM and high risk of ASCVD	9340	Composite of death from cardiovascular causes, nonfatal myocardial infarction, or nonfatal stroke	Significant reduction in primary outcome (hazard ratio, 0.87; 95% confidence interval [CI], 0.78 to 0.97)				More frequent GI side effects with IMP
EXSCEL Holman et.al.	2017	Exenatide	T2DM	14752	Composite of death from cardiovascular causes, nonfatal myocardial infarction, or nonfatal stroke	No significant differences between IMP and Placebo (hazard ratio, 0.91; 95% confidence interval [CI], 0.83 to 1.00)				No significant differences
Harmony Outcomes Hernandez et.al.	2018	Albiglutide	T2DM with established ASCVD	9463	Composite of Cardiovascular death, MI, or stroke	Significant reduction in primary outcomes with Albiglutide (hazard ratio 0·78, 95% CI 0·68–0·90)				No significant differences
REWIND Gerstein et.al.	2019	Dulaglutide	T2DM and high risk of or established ASCVD	9901	Composite of death from cardiovascular causes, nonfatal myocardial infarction, or nonfatal stroke	Reduction in primary outcome with Dulaglutide (hazard ratio [HR] 0·88, 95% CI 0·79–0·99; p=0·026)				More frequent GI side effects with IMP
PIONEER 6 Husain et.al.	2019	Oral Semaglutide	T2DM and high risk of ASCVD	3183	Composite of death from cardiovascular causes, nonfatal myocardial infarction, or nonfatal stroke	No significant reduction in primary outcome (hazard ratio, 0.79; 95% confidence interval [CI], 0.57 to 1.11). However, a significant reduction in cardiovascular and all-cause mortality noted				More frequent GI side effects with IMP
AMPLITUDE-O Gerstein et.al.	2021	Efpeglenatide	T2DM with established ASCVD or CKD and additional risk factors	4076	Composite of death from cardiovascular causes, nonfatal myocardial infarction, or nonfatal stroke	Reduction in the primary outcome with Efpeglenatide (hazard ratio, 0.73; 95% confidence interval [CI], 0.58 to 0.92)			Yes	More frequent GI side effects with IMP
SELECT Lincoff et.al.	2023	Semaglutide	Established ASCVD with BMI≥27Kg/m^2^, without Diabetes	17604	Composite of death from cardiovascular causes, nonfatal myocardial infarction, or nonfatal stroke	Significant reduction in Primary outcome (hazard ratio, 0.80; 95% confidence interval, 0.72 to 0.90)				More frequent GI side effects with IMP resulting in permanent discontinuation of IMP in a significant proportion of patients
FLOW Perkovic et.al.	2024	Semaglutide	T2DM and Albuminuric CKD	3533	Composite of sustained 50% decline in GFR, ESRD, or death from renal or cardiovascular causes	Significant reduction in primary outcome (hazard ratio, 0.76; 95% confidence interval [CI], 0.66 to 0.88)			Yes	More frequent GI Symptoms and eye conditions with IMP
DPP4i										
SAVOR-TIMI 53 Scirica et.al.	2013	Saxagliptin	T2DM and high risk of or established ASCVD	16492	Composite of Cardiovascular death, MI or stroke.	No significant reduction in the primary outcome (hazard ratio with Saxagliptin, 1.00; 95% confidence interval [CI], 0.89 to 1.12)				Significant increase in the incidence of HHF with Saxagliptin
EXAMINE White et.al.	2013	Alogliptin	T2DM and recent ACS	5380	Composite of cardiovascular death, non-fatal MI or non-fatal stroke.	No significant reduction in primary outcome (hazard ratio, 0.96; upper boundary of the one-sided repeated confidence interval, 1.16)				No significant differences
TECOS Green et.al.	2015	Sitagliptin	T2DM with established ASCVD	14671	Composite of cardiovascular death, non-fatal MI, non-fatal stroke or HUA	No significant reduction in primary outcome (Hazard ratio, 0.98; 95% CI, 0.88 to 1.09)				No significant differences
CARMELINA Rosenstock et.al.	2018	Linagliptin	T2DM with ASCVD and CKD	6991	Composite of cardiovascular death, non-fatal MI or non-fatal stroke.	No significant reduction in primary outcome (HR, 1.02; 95% CI, 0.89-1.17)				No significant differences
DPP4 vs SU										
CAROLINA Rosenstock et.al.	2019	Linagliptin vs Glimepiride	T2DM and high risk of or established ASCVD	6042	Composite of cardiovascular death, non-fatal MI or non-fatal stroke.	No significant difference with regard to the primary outcome (Hazard Ratio, 0.98 [CI], 0.84-1.14)				More hypoglycaemic events with Glimepiride
Insulin										
ORIGIN Gerstein et.al.	2012	Insulin glargine	Cardiovascular risk factors and impaired fasting glucose, impaired glucose tolerance, or T2DM	12537	Composite of cardiovascular death, non-fatal MI or non-fatal stroke and revascularisation or HHF	No significant reduction of the primary outcome (hazard ratio, 1.02; 95% confidence interval [CI], 0.94 to 1.11)				Increased incidence of hypoglycaemic events and weight gain with insulin glargine
DEVOTE Marso et.al.	2017	Insulin Degludec versus Glargine	T2DM	7637	Composite of cardiovascular death, non-fatal MI or non-fatal stroke.	No significant reduction in the primary outcome (hazard ratio, 0.91; 95% confidence interval, 0.78 to 1.06)				Degludec was associated with fewer hypoglycaemic events than glargine
Metformin VS SU / Insulin										
UKPDS Holman et.al.	1998	Metformin vs SU vs Insulin vs Diet	T2DM	4075	Major cardiovascular outcomes, MI and all-cause death	Reduction in diabetes-related end points, MI and death from any cause with Metformin				Increased risk of hypoglycaemia with intensive therapy
SPREAD-DIMCAD Hong et.al.	2013	Metformin vs Glipizide	T2DM with established ASCVD	304	Composite of cardiovascular or all-cause death, nonfatal MI, nonfatal stroke, or arterial revascularization.	Reduction in primary outcome with metformin (Hazard ratio (HR) of 0.54 (95% CI 0.30–0.90)				No significant differences
Thiazolidinediones										
PROactive Dormandy et.al.	2005	Pioglitazone	T2DM with established ASCVD	5238	Composite of All-cause death, non-fatal MI, Stroke, ACS, coronary or peripheral vascular intervention or above-ankle amputation	No significant reduction in the primary outcome (HR 0•90, 95% CI 0•80-1•02), although reduction in all-cause mortality nonfatal MI and stroke were noted				Higher incidence of HHF with Pioglitazone
RECORD Home et.al.	2009	Rosiglitazone	T2DM	4447	Composite of cardiovascular hospitalisation or cardiovascular death	No significant reduction in the primary outcome (HR 0·99, 95% CI 0·85–1·16)				Higher incidence of HHF and limb fractures in women with Pioglitazone
Thiazolidinediones vs SU										
TOSCA.IT Vaccaro et.al.	2017	pioglitazone vs SU	T2DM with inadequate glycaemic control	3028	Composite of All-cause death, non-fatal MI, non-fatal stroke or urgent coronary intervention	No significant reduction in the primary outcome (hazard ratio 0·96, 95% CI 0·74–1·26)				No significant differences
Tirzepatide										
SURPASS‐CVOT Nicholls et.al.	Ongoing	Tirzepatide vs Dulaglutide	T2DM and established ASCVD with BMI≥25 Kg/m^2^	13299	Composite of cardiovascular death, MI, or stroke	Ongoing				

Summary of major outcome trials based on various glucose-lowering agents. ACS, Acute coronary syndrome; ASCVD, Atherosclerotic cardiovascular disease; BMI, Body mass index; CV, Cardiovascular; CKD, Chronic kidney disease; DKA, Diabetic ketoacidosis; DPP4i, dipeptidyl peptidase 4 inhibitor; eGFR, estimated glomerular filtrate; EF, Ejection fraction; EMPACT-MI, Empagliflozin after Acute Myocardial Infarction; GI, Gastrointestinal; GLP1RAs, Glucagon-like peptide-1 receptor agonist; HHF, Hospitalisation for heart failure; HUA, Hospitalisation for Unstable Angina; IMP, Investigational medicinal product; KCCQ, Kansas City Cardiomyopathy Questionnaire; MI, Myocardial infarction; SCORED, Sotagliflozin in Patients with Diabetes and Chronic Kidney Disease; SU, Sulfonylurea; T2DM, Type 2 diabetes mellitus; VERTIS CV, Cardiovascular Outcomes with Ertugliflozin in Type 2 Diabetes. Colour coding key for primary inclusion criteria: Purple: ASCVD, Black: Renal, Brown: Heart failure, Blue: MI.

### 3.1 Lifestyle modification

European Society of Cardiology (ESC) guidelines highlight the integral role that lifestyle modifications play in the overall management of T2DM in those with underlying ischemic heart disease. It recommends a multi-faceted approach to address both weight loss and dietary changes, with increased routine physical activity to enhance benefits. Such changes not only help control diabetes but also may result in improvement in blood pressure control as well. More than 5% weight loss is known to improve glycaemic control in addition to lipid and blood pressure control in individuals who are obese (10, 33). Structured weight loss programs and medical therapy, including the use of GLP-1 receptor agonists, may prove helpful in addressing obesity. High-risk individuals with persistently elevated body mass index (BMI) greater than 35 Kg/m^2^ may benefit from bariatric surgery (10). Similarly, the Mediterranean diet has also shown to be beneficial with a shift from animal to plant-based products. Alcohol should be taken in moderation as well as reducing saturated fats and food intake (10, 34). Additionally, sodium intake should be reduced to 2.5 g/day which is also shown to decrease systolic blood pressure by about 5.8mmHg in individuals with pre-existing hypertension (10). Regular exercise is recommended with at least 150 min of moderate weekly activity. Structured programs can be helpful and may result in a reduction of HbA1c by almost 0.6% via a combination of resistance and endurance training (10, 35). Smoking cessation advice should be provided to all patients as this alone can result in a reduction of 36% mortality in those with cardiovascular disease irrespective of diabetes mellitus. Adjuvant therapies to help with this may include the prescription of nicotine replacement therapy in the form of gums or transdermal patches (10, 34). All of these factors need to be addressed and discussed with the patient in a multidisciplinary fashion to ensure compliance in the long term for maximum benefits (10).

### 3.2 Glucose-lowering medications with proven cardiovascular benefits

#### 3.2.1 Sodium-glucose co-transporter 2 inhibitors

Several large randomised controlled trials (RCTs) have shown cardiovascular benefits associated with SGLT2 inhibitors in individuals with T2DM and ischemic heart disease. The EMPA-REG OUTCOME trial (Empagliflozin, cardiovascular outcomes and mortality in type 2 diabetes mellitus- reducing excess glucose) showed a significant reduction in all-cause mortality, cardiovascular death, and hospitalisation for heart failure (HHF) in those receiving Empagliflozin (12, 36). This was followed by the CANVAS trial (Canagliflozin and cardiovascular and renal events in type 2 diabetes) showing reduction with Canagliflozin in the primary composite outcome of death from cardiovascular causes, nonfatal myocardial infarction (MI) and nonfatal stroke (13) and the DECLARE TIMI 58 trial (Dapagliflozin and cardiovascular outcomes in type 2 diabetes) where Dapagliflozin therapy resulted in a significant reduction in cardiovascular death and HHF (14). In contrast, the VERTIS CV (Evaluation of Ertugliflozin efficacy and safety cardiovascular outcomes trial in type 2 diabetes) trial did not reflect similar benefits with Ertugliflozin, nonetheless it continued to show that SGLT2 inhibitors were safe in these individuals (37). Real-world data has reflected the same benefits in those with T2DM when receiving an SGLT2 inhibitor in comparison to other glucose-lowering medications (38).

Sodium-glucose co-transporter 2 inhibitors offer additional benefits in terms of heart failure events and renal outcomes reduction. The SCORED trial (Sotagliflozin in patients with diabetes and chronic kidney disease) assessed Sotagliflozin in individuals with known chronic kidney disease (CKD) and T2DM with regards to cardiovascular outcomes and noted a significant reduction in the composite outcome of cardiovascular death and heart failure (39). Similarly, Canagliflozin in the CREDENCE trial (Canagliflozin and kidney-related adverse events in type 2 diabetes and CKD) showed a reduction in the incidence of serious and non-serious kidney-related events in those with diabetes and CKD (17, 40). Other RCTs, systematic reviews and meta-analyses have shown similar results (18, 41–44), whilst another systemic review and meta-analysis failed to show beneficial or adverse effects of ischaemic stroke reductions with SGLT2 inhibitors, although it did identify benefits against haemorrhagic stroke (45).

Initial unexpected results in cardiovascular outcome trials and the benefits seen with a reduction in HHF paved the way for other dedicated heart failure trials. The DAPA HF (Dapagliflozin in patients with heart failure and reduced ejection fraction), EMPEROR-Reduced (cardiovascular and renal outcomes with Empagliflozin in heart failure) and SOLOIST-WHF (Sotagliflozin in patients with diabetes and recent worsening heart failure) trials showed a significant reduction in the risk of worsening heart failure or death from cardiovascular causes among those taking SGLT2 inhibitors in patients with heart reduced ejection fraction (46–48). These were followed by DELIVER (Dapagliflozin in Heart Failure with Mildly Reduced or Preserved Ejection Fraction) and EMPEROR-Preserved (Empagliflozin in heart failure with a preserved ejection fraction) trials which showed similar benefits in patients with mildly impaired or preserved ejection fraction (49, 50) and meta-analysis highlighting reduced risk of cardiovascular death and worsening heart failure (51, 52). Two trials assessed starting Empagliflozin in patients with acute heart failure which also showed a reduction in re-hospitalisation for heart failure or death at 60 to 90 days and a satisfactory safety profile (53, 54).

The possible benefits of initiating SGLT2i therapy in individuals with acute coronary syndrome and T2DM are still unclear due to a lack of dedicated randomised controlled trials in this cohort of patients. The EMPACT-MI (Empagliflozin after acute myocardial infarction) aimed to assess this in patients with or without T2DM when started within 14 days of admission. Treatment with Empagliflozin did not lead to a significant reduction of the composite outcome of first HHF or death from any cause. However, it should be noted that only around 32% of patients had an underlying diagnosis of T2DM, and therefore, were likely to be underpowered to assess for any benefits in this group (55). Further *post-hoc* analysis would be helpful in highlighting potential benefits and formulating additional hypotheses. The DAPA MI trial (Dapagliflozin in myocardial infarction without diabetes or heart failure) excluded patients with diabetes and was a neutral trial concerning hard outcomes of cardiovascular death or hospitalization for heart failure (HHF) (56). In contrast, real-world data from a national registry of patients with T2DM admitted with ACS showed a significant reduction in composite outcome of all cause death and HHF with SGLT2is when prescribed at discharge in comparison to those that didn’t (hazard ratio [HR] 0.70, 95% confidence interval [CI] 0.59–0.82). However, this was an observational study with potential unaddressed underlying biases that may have been present between the two groups (21). Moreover, patients were divided into groups at the time of the index event and it was unclear whether or not those in non-SGLT2i group may have subsequently been commenced on the therapy, thereby blunting the true findings between those that didn’t receive the medicine in comparison to those that did. Individuals with diabetes are inherently at a higher risk of further cardiovascular events particularly in the context of an ACS, and additional dedicated studies would be needed to evaluate benefits versus the risks from early initiation of SGLT2 inhibition in those with T2DM especially in the setting of acute coronary syndromes.

In summary, SGLT2 inhibitors provide multiple benefits in individuals with T2DM and IHD, primarily via a reduction in cardiovascular death, all-cause death, and renal and hospitalisation for heart failure events. These benefits are also reflected in international guidelines from the ESC, American Diabetes Association (ADA), and the European Association for the Study of Diabetes (EASD), all recommending use in these patients (10, 57).

#### 3.2.2 Glucagon-like peptide-1 receptor agonists

Glucagon-like peptide-1 (GLP-1) receptor agonists reduce blood glucose and improve postprandial metabolism. Additionally, they stimulate hypothalamic neurons to evoke satiety, helping weight loss. GLP-1 receptor agonists were initially developed as a glucose-lowering medication (58). Initial large RCTs demonstrated good safety profiles with subsequent trials showing additional cardiovascular benefits irrespective of HbA1c level and diabetes control (10, 59). The ELIXA (Lixisenatide in patients with type 2 diabetes and acute coronary syndrome) trial showed a good safety profile for Lixisenatide use in those with T2DM following a recent ACS, although there was no significant difference in major cardiovascular events between the two arms (60). Similar results were also seen for the EXSCEL (effects of once-weekly Exenatide on cardiovascular outcomes in type 2 diabetes) trial (61). Further studies, including LEADER (Liraglutide and cardiovascular outcomes in type 2 diabetes) (15), SUSTAIN-6 (Semaglutide and cardiovascular outcomes in patients with type 2 diabetes) (16), REWIND (Dulaglutide and cardiovascular outcomes in type 2 diabetes) (62), Harmony Outcomes (Albiglutide and cardiovascular outcomes in patients with type 2 diabetes and cardiovascular disease) (63) and AMPLITUDE-O (cardiovascular and renal outcomes with Efpeglenatide in type 2 diabetes) (64) trials all showed significant reduction in cardiovascular events. Only one form of oral GLP-1 receptor agonist (Semaglutide) is available and licensed at present. It had a good safety profile when trialed in the PIONEER 6 (oral Semaglutide and cardiovascular outcomes in patients with type 2 diabetes) study and showed a reduction in death from cardiovascular and death from any causes with oral Semaglutide, however, overall major adverse cardiovascular events did not differ among the two arms (65). The above-mentioned trials were all carried out in individuals with known T2DM and who had a previous history of IHD or who were at high risk of having an event. Meta and *post-hoc* analysis of these trials showed an overall significant reduction in all cardiovascular, cerebrovascular, and kidney events and mortality in this group of patients (66, 67).

More recently, data suggests that GLP-1 receptor agonists may have additional benefits. The FLOW (effects of Semaglutide on chronic kidney disease in patients with type 2 diabetes) trial showed a significant reduction in kidney outcomes and cardiovascular death with Semaglutide in those with T2DM and CKD (20). Similarly, in the SELECT (Semaglutide and cardiovascular outcomes in obesity without diabetes) trial, the same GLP-1 receptor agonist when administered to overweight or obese individuals with pre-existing cardiovascular conditions resulted in a reduction in the incidence of cardiovascular death, non-fatal MI, or nonfatal stroke (68) as well as a reduction in symptoms and physical limitations in obese individuals with heart failure and preserved ejection fraction (69).

Based on the evidence provided by these trials and meta-analysis, the ESC, ADA, and EASD guidelines recommend initiation of GLP-1 receptor agonists with proven benefits, irrespective of diabetes control, in patients with cardiovascular disease and T2DM (10, 59).

#### 3.2.3 SGLT2 inhibitors and GLP-1 receptor agonist combination therapy

A meta-analysis of the RCTs with regards to both classes of medications showed beneficial effects in comparison to other glucose-lowering drugs (70, 71) with one study suggesting reno-cardiovascular benefits of SGLT2 inhibitors irrespective of background GLP-1 receptor agonist therapy (72). A recent population-based cohort study has also shown a greater reduction in cardiovascular and renal events with a combination of SGLT2 inhibitors and GLP-1 receptor agonists in comparison to either agent given alone (73). While SGLT2 inhibitors appear superior with regards to reduction in HHF and renal events, GLP-1 receptor agonists appeared to significantly reduce the risk of stroke. Therefore, the benefits of combination therapy are likely to be complementary to each other and recommendations are to be individualised to patients with respect to existing co-morbid conditions and availability of medications with proven efficacy (58, 70, 71, 74–76).

### 3.3 Glucose-lowering agents with doubtful or no cardiovascular benefit

#### 3.3.1 Metformin

The United Kingdom Prospective Diabetes Study (UKPDS) was the first study in which intensive blood glucose control with either insulin, sulphonylurea, or metformin, was assessed versus diet control in patients with T2DM. The study showed that metformin appeared to decrease the risk of diabetes-related endpoints in overweight diabetic patients with less weight gain and a lower number of hypoglycaemic attacks in comparison to insulin (77). Since then, Metformin has been considered to be the first-line glucose-lowering therapy for those with T2DM. A 10-year follow-up of the same study continued to show a reduction in the microvascular risk as well as a reduction in MI and all-cause death (78). Another study looked at metformin versus sulphonylurea glipizide in individuals with T2DM and IHD and noted substantially reduced major cardiovascular events in the Metformin (79). However, most of these trials either had a small number of patients, few cardiovascular events or lacked of head-to-head comparison with other glucose-lowering medications (10). Subsequent meta-analyses concluded clinical uncertainty of any significant reduction of cardiovascular outcomes from metformin use in those with T2DM, over and above that expected with a reduction in glucose levels (80). With the introduction of SGLT2 inhibitors and GLP-1 receptor agonists, the benefits of metformin have become even less pronounced (81). Another meta-analysis, including six trials and 51,743 participants, assessed SGLT2 inhibitors with and without metformin and showed that the former reduced the risk of major adverse cardiovascular events (MACE), irrespective of concomitant metformin therapy (82). Therefore, based on the limitations above, the latest ESC guidelines recommend metformin as a second-line agent, after the introduction of SGLT2 inhibitors or GLP-1 receptor agonists, primarily as a glucose-lowering agent without any significant cardiovascular risk reduction benefits (10). Similar recommendations are also provided by the ADA and EASD guidelines as well (59).

#### 3.3.2 Dipeptidyl peptidase 4 inhibitors

Dipeptidyl peptidase (DPP)-4 inhibitors prevent the rapid degradation of glucagon-like peptide 1 through the inhibition of DPP-4 receptors, thereby enhancing pancreatic insulin and suppressing glucagon secretion resulting in a reduction in blood sugar levels (83). The first major RCT for DPP-4 inhibitors, the EXAMINE (Examination of Cardiovascular Outcomes with Alogliptin versus Standard of Care) trial assessed cardiovascular outcomes with Alogliptin in patients with T2DM who had a recent ACS. The trial concluded non-inferiority of Alogliptin in comparison to placebo, however, numerically greater, but non-significant, heart failure events were noted (10, 83). Another parallel study RCT used Saxagliptin in patients with T2DM who had a history of or were at increased risk of cardiovascular events. The findings suggested a significant increase in hospitalisation for heart failure events with the DPP-4 inhibitor (84, 85). However, in the TECOS (effect of Sitagliptin on cardiovascular outcomes in type 2 diabetes) trial, Sitagliptin appeared to be safe and did not result in an increased risk of major cardiovascular events including HHF (86, 87) with similar results with Linagliptin from the CARMELINA (effect of Linagliptin vs placebo on major cardiovascular events in adults with type 2 diabetes and high cardiovascular and renal risk) trial (88, 89). Linagliptin has further been compared with Glimepiride (90) as well as Insulin glargine (91), and was noted to be non-inferior with regards to cardiovascular outcomes and with a significantly reduced incidence of significant hypoglycaemia. Therefore, while some DPP-4 inhibitors such as Saxagliptin may cause worsening of heart failure events, Sitagliptin, and particularly Linagliptin has significant clinical data available to suggest a good safety profile, albeit with no significant cardiovascular benefits. Therefore, specific cardiovascular neutral DPP-4 is may be recommended as adjuvant therapy to further optimise diabetes control in those with IHD (10).

#### 3.3.3 Thiazolidinediones

Thiazolidinediones are peroxisome proliferator-activated receptor-gamma (PPAR-gamma) agonists which improve insulin sensitivity and thereby not only reduce blood glucose levels but also have anti-inflammatory properties. The PROACTIVE (PROspective pioglitAzone Clinical Trial In macroVascular Events) study was one of the first trials to assess this class of medicines for cardiovascular outcomes in patients with T2DM and didn’t report any significant differences for the primary outcomes between the two arms. However, a key secondary outcome composite of all-cause mortality, non-fatal MI, and non-fatal stroke was significantly reduced with Pioglitazone at the cost of an increased incidence of admission with hospitalization for heart failure(HHF) (92). This was likely due to increased fluid retention and expanded plasma volume, resulting in HHF events (10). Reduction in ischemic stroke events and transient ischaemic attacks (TIAs) was also noted with Pioglitazone in non-diabetic patients (93) as well as a reduction in major cardiovascular events and death in patients with T2DM and end-stage renal disease (ESRD) (94). However, similar to the PROACTIVE trial, it was associated with an increased risk of weight gain, oedema, and fractures (93, 95), with the above findings further confirmed in multiple meta-analyses of the major trials (96–98). Similar findings were also noted with Rosiglitazone (99–101).

Therefore, even though the above evidence may suggest a reduction in cardiovascular events such as stroke with this class of medications, any benefits are offset by a significant increase in heart failure events and increased risk of fractures in females. With the introduction of newer agents with better safety profiles, these should be avoided if possible (10).

#### 3.3.4 Insulin

Insulin remains a key intervention in the management of T2DM, especially in those whose control remains poor despite multiple therapeutic agents. Due to the rapid onset of action, adjustable dosage, and potent glucose-lowering effects, it may be an effective intervention to manage uncontrolled hyperglycaemia in patients with ACS, with normalisation of blood glucose levels known to improve outcomes (102). Insulin glargine has proven to be safe with no significant adverse effects on cardiovascular outcomes in cancer, although it was associated with an increased incidence of hypoglycaemia and weight gain (103). Similarly Degludec, an ultra-long-acting insulin, also proved safe with respect to major cardiovascular events (104). Therefore, insulin may be considered as an option for improving diabetes control after the initiation of other therapies with established cardiovascular benefits in situations where optimum diabetes control has not been achieved previously (10). However, care has to be taken in these patients as insulin does not seem to offer additional benefit when glucose levels are well controlled (105), in contrast hypoglycaemic events and intensive glucose lowering particularly in the context of ACS were associated with increased mortality (23, 106).

#### 3.3.5 Sulphonylureas

Sulfonylureas were one of the earlier classes of medications used to treat T2DM. They are commonly available and associated with low cost (107). Although there have been no head-to-head randomised controlled studies purely on sulphonylureas, there have been multiple studies combined with other glucose-lowering drugs to assess their utility and benefits. Sulphonylureas reduce microvascular complications of T2DM, probably due to improved diabetes control (78, 108). A nationwide registry study compared sulfonylureas with metformin in patients with T2DM and noted that monotherapy with sulfonylureas seemed to be associated with increased mortality and cardiovascular risk compared to metformin. However, among the various sulphonylureas used in the study, Gliclazide and Repaglinide appeared safer than the rest and were not associated with a significantly increased risk in comparison to metformin (109). A subsequent RCT assessing Glimepiride and Gliclazide versus Pioglitazone as add-on treatments to metformin showed a good overall safety profile between the two arms of the study, although the former was associated with a slightly higher incidence of hypoglycemia (110). Similar results were noted compared with Linagliptin (90). Sulfonylureas are effective in glycemic reduction (111), and represent a reasonable add-on therapy in patients with uncontrolled diabetes to further improve their glycemic index. However, care should be taken to avoid hypoglycemic events, aiming to use agents with proven safety profiles such as Glimepiride and Gliclazide (10), particularly at the time of and in the acute months after ACS, which is associated with increased mortality (23, 106).

#### 3.3.6 Dual GIP/GLP-1 receptor agonist

Dual glucose-dependent insulinotropic polypeptide (GIP) and glucagon-like peptide 1 (GLP-1) receptor agonists have recently been developed for the management of T2DM. Tirzepatide has recently been approved for this purpose and its safety and efficacy have been assessed in multiple RCTs, where results suggest a significant reduction in HbA1c as well as body weight in comparison to placebo (112), Semaglutide (113), insulin degludec (114), insulin Glargine (115, 116) and similar safety profile to GLP- receptor agonists and a lesser incidence of hypoglycemia in comparison to insulin or sulfonylureas in the above studies. These findings have been further analysed in a meta-analysis showing dose-dependent superiority for glycemic control and body weight reduction with similar results as above without any increased risk of major cardiovascular events (117), although an increased incidence of gastrointestinal adverse effects (118) was noted with the Tirzepatide. The SURPASS-CVOT (comparison of Tirzepatide and Dulaglutide on major adverse cardiovascular events in participants with type 2 diabetes and atherosclerotic cardiovascular disease) is an ongoing trial aiming to assess for any additional cardiovascular outcome benefits over and above current standard treatment with GIP/GLP-1 receptor agonists (119). For now, it represents a feasible option in patients with suboptimal diabetes control despite multiple agents, especially those who are overweight or obese and in whom a significant degree of HbA1c reduction and weight loss is required (10).

## 4 Conclusion and future work

**Figure 2:**
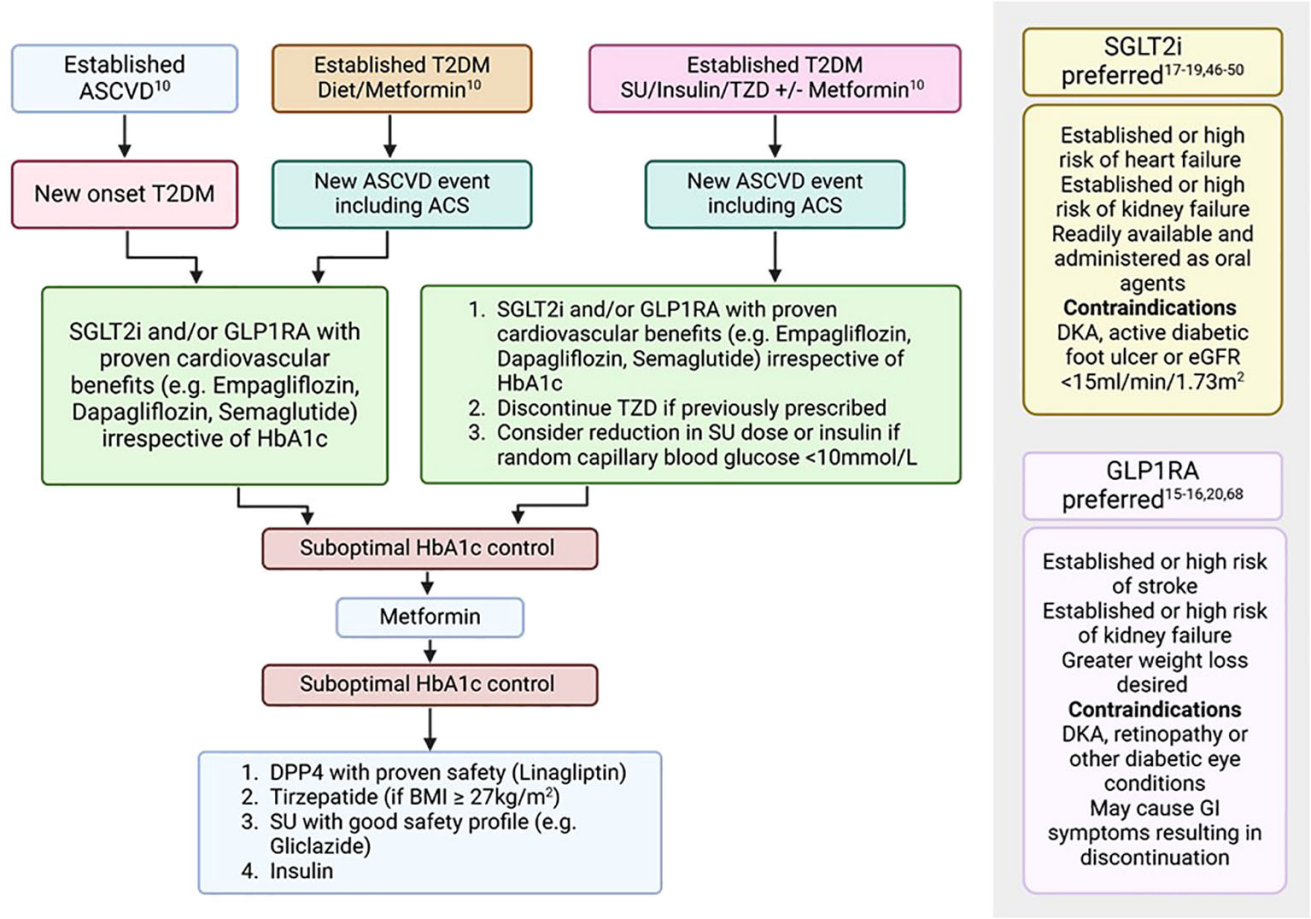
Treatment algorithm for the management of T2DM in patients with ASCVD (10, 15–20, 46–50, 68). ASCVD, Atherosclerotic cardiovascular disease; BMI, Body mass index; DKA, Diabetic ketoacidosis; eGFR, estimated glomerular filtration rate; GI, Gastrointestinal; GLP1RA, Glucagon-like peptide-1 receptor agonists; SGLT2i, Sodium-glucose co-transport-2 inhibitors; SU, Sulfonylurea; T2DM, Type 2 diabetes mellitus; TZD, Thiazolidinediones.

Type 2 diabetes mellitus remains a significant risk factor for the development of IHD and timely diagnosis and management is paramount to improve clinical outcomes. Individuals with chronic and acute coronary syndromes must be screened regularly for T2DM using HbA1c and random blood glucose assessments. In cases of inconclusive results, OGTT may be further performed for the diagnosis of the condition. Due to ease of use, HbA1c is also recommended for monitoring of T2DM whilst Fructosamine is to be performed in those patients with additional co-morbid conditions such as haemoglobinopathies. If not contraindicated, SGLT2is and GLP1RAs should be considered first-line treatment irrespective of HbA1c levels in those with established IHD (**Figure 2**, **Table 2**). Subsequently, additional medications such as Metformin, DPP4is and dual GIP/GLP-1 receptor agonists may be further considered to optimise diabetes control (**Figure 2**, **Table 2**). Evidence for the use of glucose-lowering therapies with proven cardiovascular benefits, especially SGLT2is, in an ACS setting, is limited to mostly non-diabetic population. However, a Swedish nationwide registry study suggests significant benefits when initiated immediately after an acute coronary event in patients with T2DM (21). Further studies need to be performed to not only assess any benefits of use in such acute settings but also to assess for optimal timing of initiation of such therapies to improve outcomes and reduce unintended complications. As the burden of diabetes continues to rise, clinicians specialising in diabetes and cardiology need to be cognisant of the latest developments in this dynamic field. This will promote early change of practice with regards to managing these highly complex conditions, allowing for improved outcomes, and a reduction in burden on our healthcare system.
